# Impact of Blood Sample Collection and Processing Methods on Glucose Levels in Community Outreach Studies

**DOI:** 10.1155/2013/256151

**Published:** 2013-01-10

**Authors:** Michael Turchiano, Cuong Nguyen, Arthur Fierman, Mark Lifshitz, Antonio Convit

**Affiliations:** ^1^Department of Psychiatry, New York University School of Medicine, New York, NY 10016, USA; ^2^Department of Pediatrics, New York University School of Medicine, New York, NY 10016, USA; ^3^Department of Pathology and Medicine, New York University School of Medicine, New York, NY 10016, USA; ^4^Nathan Kline Institute for Psychiatric Research, Orangeburg, NY 10962, USA; ^5^Brain, Obesity, and Diabetes Laboratory (BODyLab), New York University School of Medicine, 145 East 32nd Street, 8th Floor, New York, NY 10016, USA

## Abstract

Glucose obtained from unprocessed blood samples can decrease by 5%–7% per hour due to glycolysis. This study compared the impact of glucose degradation on measured glucose values by examining two different collection methods. For the first method, blood samples were collected in tubes containing sodium fluoride (NaF), a glycolysis inhibitor. For the second method, blood samples were collected in tubes containing a clot activator and serum gel separator and were centrifuged to separate the serum and plasma 20 minutes after sample collection. The samples used in the two methods were collected during the same blood draw and were assayed by the clinical laboratory 2–4 hours after the samples were obtained. A total of 256 pairs of samples were analyzed. The average glucose reading for the centrifuged tubes was significantly higher than the NaF tubes by 0.196 ± 0.159 mmol/L (*P* < 0.01) or 4.2%. This study demonstrates the important role collection methods play in accurately assessing glucose levels of blood samples collected in the field, where working environment may be suboptimal. Therefore, blood samples collected in the field should be promptly centrifuged before being transported to clinical labs to ensure accurate glucose level measurements.

## 1. Introduction

The childhood obesity epidemic has been accompanied by an increase in children with impaired fasting glucose, impaired glucose tolerance, metabolic syndrome, and type 2 diabetes [1, 2]. The diagnoses of these conditions in children depend on reliable blood glucose determinations. It is important to recognize that the preanalytical handling of blood samples intended for glucose measurement can influence the laboratory results [3]. When left unprocessed, glycolysis occurs in the cellular component of a blood sample and may consume 5%–7% of the sample's glucose content per hour [4]. This is particularly relevant while conducting community or public health efforts, where the blood samples are collected in the field and several hours may elapse from time of collection to laboratory analysis.

There are numerous publications detailing a variety of handling methods that can reduce loss of glucose [5]. The American Diabetes Association suggests prompt placement of blood samples in an ice slurry or immediate separation of plasma from blood cells can halt glycolysis [6]. However, such practice may not always be possible when transporting samples from the field to the clinical laboratory. A widely used technique involves the addition of NaF to blood tubes. NaF inhibits enolase, an enzyme in the glycolytic pathway. Although NaF has been shown to completely arrest glycolysis by four hours, it has little to no effect on the rate of glycolysis during the first 1-2 hours [7]. Gambino et al. further demonstrated that acidification of blood samples using a combination of citrate buffer, NaF, and EDTA was more effective in arresting glycolysis from the moment of blood collection; however, tubes with this combination are not commercially available in the United States [8]. Collection in tubes with a clot activator, serum gel separator, and prompt centrifugation separates serum from the cellular component thereby stopping glycolysis. 

This patient-independent variability in glucose levels highlights the importance of considering these collection methods and establishing suitable protocols, particularly in community outreach programs. We sought to determine how two different blood collection and processing methods impacted fasting glucose levels in the same set of participants. The purpose of this study was to directly assess the impact of different collection methods on measured glucose levels in a nonclinical environment. Furthermore, we explored whether the glucose values obtained by the two methods affected the resulting clinical diagnoses of impaired fasting glucose and insulin resistance. Impaired fasting glucose (IFG) (≥5.55 mmol/L) has been used to estimate glucoregulatory control [9], and as has been previously described in adolescent populations, depending on the populations studied, the HOMA-IR value that has been used to define insulin resistance has varied across studies [10, 11]. 

## 2. Methods

The data came from a subsample of The Banishing Diabetes and Diabetes in Youth (BODY) Project, a school-based health screening and education program that is part of NYU Langone Medical Center's Community Service Plan, which is described in detail elsewhere [12]. Participants were asked to arrive between 7:30 AM and 8:30 AM after a 10–12 hour overnight fast. The study was approved by the institutional review boards of the New York University School of Medicine, the New York City Department of Education, the New York City Department of Health and Mental Hygiene, and the Nathan Kline Research Institute. 

### 2.1. Blood Chemistry Measurements

For a subset of participants two different glucose determinations were made based on samples obtained at the same time but collected and handled differently. The first sample was collected in a tube containing sodium fluoride (NaF tube) and placed on ice in a cooler. The second sample was collected in a tube with a clot activator and serum gel separator, then centrifuged at 3000 rpm for 10 minutes within 20 minutes of blood draw, and finally placed on ice. Both samples were transported to the clinical laboratory in a cooler with an ice block within 2–4 hours of being drawn, and the plasma glucose concentration was measured in mmol/L by the glucose oxidase method (VITROS 5600, Ortho Clinical Diagnostics, Rochester, NY). Please note that to conduct this study no additional blood samples were necessary. We simply centrifuged a tube that was being collected to measure the cholesterol profile at the field site and used an aliquot from this spun tube to contrast it to the standard NaF tube used to collect the glucose sample.

For each subject a single plasma insulin level was assayed from the centrifuged tube with the Abbott Architect (Abbott Diagnostics, Abbott Park, Illinois). From the glucose and insulin determinations, HOMA-IR was calculated as follows: fasting blood glucose (mmol/L) × fasting insulin (microIU/mL)/22.5 [13]. All blood samples (including the paired samples for glucose determination) were assayed by the clinical laboratory 2–4 hours after the venous blood samples were obtained. Although somewhat arbitrary, but based on prior studies, including one from our laboratory, we used a HOMA-IR value of ≥3.99 to define an adolescent as having significant insulin resistance [11, 14]. 

### 2.2. Statistical Analyses

Comparisons between the means were computed using paired sample and independent sample *t*-tests where appropriate. All analyses were conducted using SPSS version 19. Statistical significance was set at *α* ≤ 0.05.

## 3. Results

The study sample included 256 nondiabetic healthy students with an average age of 16.5 years (range was 14 to 18 years), height 1.68 m (1.44 to 1.94 m), weight 68.2 kg (40.37 to 153.86 kg), and body mass index 24.0 kg/m^2^ (16.0 to 48.7 kg/m^2^) with 42% female. 

Glucose values from the centrifuged tube were consistently and significantly higher than those from the tube preserved with NaF. The plasma glucose concentration in the centrifuged tubes was 4.70 mmol/L (range was 3.89 to 5.83 mmol/L) and in the NaF tubes was 4.50 mmol/L (3.72 to 5.72 mmol/L; *P* < 0.01). The average difference between the two glucose readings was 0.20 mmol/L (95% CI was 0.18 to 0.22 mmol/L; *P* < 0.01) or 4.2%. 

The number of participants identified with IFG using the centrifuged tubes was 4% or 1.56%, and the number identified with NaF tubes was 1% or 0.39%. A paired sample *t*-test did not show significance in the number identified with IFG with the two processing methods. HOMA-IR values differed significantly by the blood collection method used, with the values utilizing the centrifuged sample showing higher levels. The calculated HOMA-IR value for the centrifuged samples was in average 2.29 (range was .41 to 8.34) and for the NaF sample 2.19 (range was .39 to 7.43; *P* < 0.01). The mean HOMA-IR difference between the centrifuged tubes and NaF tubes was 0.097 (95% CI was 0.083 to 0.111; *P* < 0.01). The number of participants identified with HOMA-IR ≥3.99 using the centrifuged tubes was 24% or 9.38% and the NaF tubes was 22% or 8.59%. The average fasting insulin level of those with HOMA-IR ≥3.99 was 23.1 microIU/mL (range 17 to 35 microIU/mL) for the centrifuged tubes and 23.4 microIU/mL (17 to 35 microIU/mL) for the NaF tubes. The differences between mean glucose values and HOMA-IR scores between the two collection methods are shown in Table 1. 

## 4. Discussion

This paper highlights the challenges of measuring glucose levels outside of a highly controlled clinical setting. Waring et al. studied the differences in glucose levels obtained from tubes containing NaF to samples that had undergone prompt centrifugation during a controlled clinical trial [15]. They found a relative difference of glucose values of 4.7% between the centrifuged and NaF-treated samples. We have demonstrated that the use of sodium fluoride as a stabilizer of blood glucose levels results in a statistically significant 4.2% reduction in blood glucose values when compared to those collected in a tube with a clot activator and serum gel separator and centrifuged within 20 minutes of collection. These differences in determined glucose levels resulted in statistically significant computations of HOMA-IR by the two collection methods and resulted in more subjects being classified as having IFG. However, given the young age of our population, who on average had a BMI of only 24.0 kg/m^2^, this was a very low risk population for IFG. 

 To ascertain the clinical implication of these differences in glucose measurement, we looked at the percent of participants with IFG on the two collection methods. We found that 0.39% were identified with NaF-stabilized tubes, whereas 1.56% were detected by samples centrifuged within 20 minutes of collection. Similarly, the difference in computed HOMA-IR values utilizing the two glucose determination methods only varied by 0.10 ± 0.04. However, given that the incidence of IFG among nondiabetic adolescents is extremely low, this likely has little import in this particular population, but will be very relevant in an obese and/or significantly older population at high risk for metabolic disease. 

Given the obesity epidemic in children and the high prevalence of metabolic syndrome, the importance of identifying affected children prior to the development of type 2 diabetes cannot be overemphasized. This paper demonstrates the differential impact of two differing glucose processing methodologies on measured glucose values, specifically for samples collected in the field, where several hours elapse from collection until the assays are run in the clinical laboratory. Future studies should make an effort to collect samples in tubes with a clot activator and serum gel separator and centrifuge samples on site. We have demonstrated that this is feasible even in a large metropolitan area public high school without laboratory or medical facilities.

## Figures and Tables

**Table 1:** Mean glucose and HOMA values as well as rates of IFG and MetS_IFG_ and MetS_HOMA_ for NaF-treated tube and centrifuged plain Tube.

	Centrifuged Plain Tube	NaF Tube	*P* value
Fasting glucose (mmol/L) ± SD	84.56 ± 6.42	81.02 ± 5.82	<0.01
HOMA-IR	2.29 ± 1.82	2.1 ± 1.78	<0.01
IFG (≥100 mmol/L) % (*n*)	1.56 (4)	0.39 (1)	0.083
HOMA-IR ≥3.99% (*n*)	9.38 (24)	8.59 (22)	0.158

HOMA-IR: Homeostatic model assessment of insulin resistance; IFG: impaired fasting glucose.
